# Households’ vulnerability assessment: empirical evidence from cyclone-prone area of Bangladesh

**DOI:** 10.1186/s40562-023-00280-z

**Published:** 2023-06-06

**Authors:** Md Mostafizur Rahman, Md. Saidul Islam Arif, Md. Tanvir Hossain, Hussein Almohamad, Ahmed Abdullah Al Dughairi, Motrih Al-Mutiry, Hazem Ghassan Abdo

**Affiliations:** 1 Department of Disaster Management & Resilience, Faculty of Arts and Social Sciences, Bangladesh University of Professionals, Dhaka, 1216 Bangladesh; 2 Sociology Discipline, Social Science School, Khulna University, Khulna, 9208 Bangladesh; 3 Department of Geography, College of Arabic Language and Social Studies, Qassim University, Buraydah 51452, Saudi Arabia; 4 Department of Geography, College of Arts, Princess Nourah bint Abdulrahman University, Riyadh 11671, Saudi Arabia; 5 Geography Department, Faculty of Arts and Humanities, Tartous University, Tartous, Syria

**Keywords:** Cyclone, Risk assessment, Exposure, Vulnerability, Bangladesh

## Abstract

Despite Bangladesh being vulnerable to cyclones, there is a dearth of research on cyclone vulnerability assessment. Assessing a household's vulnerability is considered a crucial step in avoiding the adverse effects of catastrophe risks. This research was conducted in the cyclone-prone district of Barguna, Bangladesh. This study's purpose is to evaluate this region's vulnerability. A questionnaire survey was conducted using a convenience sample technique. A door-to-door survey of 388 households in two Unions of Patharghata Upazila, Barguna district, was conducted. Forty-three indicators were selected to assess cyclone vulnerability. The results were quantified using an index-based methodology with a standardized scoring method. Where applicable, descriptive statistics have been obtained. In terms of vulnerability indicators, we also utilized the chi-square test to compare Kalmegha and Patharghata Union. When appropriate, the non-parametric Mann–Whitney U test was employed to evaluate the relationship between the Vulnerability Index Score (VIS) and the union. According to the results, the environmental vulnerability (0.53 ± 0.17) and the composite vulnerability index (0.50 ± 0.08) were significantly greater in Kalmegha Union than in Patharghata Union. They faced inequity in government assistance (71%) and humanitarian aid (45%) from national and international organizations. However, 83% of them underwent evacuation practices. 39% were satisfied with the WASH conditions at the cyclone shelter, whereas around half were dissatisfied with the status of the medical facilities. Most of them (96%) rely only on surface water for drinking. National and international organizations should have a comprehensive plan for disaster risk reduction that encompasses all individuals, regardless of race, geography, or ethnicity.

## Introduction

As developing and developed countries’ populations cluster around the coast, more people and properties will be at risk from tropical cyclones (Woodruff et al. 2013; Pilkington and Mahmoud 2017; Edmonds et al. 2020). Increases in global mean surface temperatures, temperature gradients, and atmospheric moisture are predicted to increase the intensity and frequency of tropical cyclones in the future (McNutt 2015; Edmonds et al. 2020; Nasir et al. 2022; Rendana et al. 2023). To lessen the impact of these cyclones in the short and long term, strategic capacity enhancement of critical infrastructure is necessary (McNutt 2015; Edmonds et al. 2020; Reddy et al. 2023). Economic losses due to natural catastrophes have risen in the South Asian region as the worldwide influence of climate extremes has grown (ESCAP 2021). Bangladesh has been ranked as the world’s thirteenth most dangerous country by the World Risk Index for 2021 (Aleksandrova et al. 2021). It is one of the most vulnerable countries in the Asian region (Aleksandrova et al. 2021). Bangladesh is in extreme peril from climatic extremes due to its geophysical conditions, climate extremes, and high vulnerability and exposure (ESCAP 2021). The coasts of Bangladesh are often impacted by cyclones, making the country a cyclone risk (Sattar and Cheung 2019; Hoque et al. 2021). Cyclones frequently form in the Bay of Bengal during the early summer (April to June) and the late rainy season (September to November), with which it shares a southern border (Paul 2009; Uddin et al. 2019). This country has a long history of cyclones (Alam et al. 2020). Numerous cyclones have caused devastation in coastal areas, killing many people and destroying a great deal of property (Hossain 2015; Alam et al. 2020). Nearly half a million and 140,000 were killed when two major cyclones landed in Bangladesh's coastal areas in 1970 and 1991 (Alam and Dominey-Howes 2015; Sattar and Cheung 2019). The 2007 cyclone Sidr killed 3500 people and cost the economy $1.67 billion (Alam et al. 2020). 2009’s Cyclone Aila killed 190 people, injured 7000 more, and destroyed over 500,000 homes (Ahmed et al. 2016). Due to the low elevation of many coastal areas, many people will be at risk from storm surges induced by rising sea levels (Rana et al. 2010; Mallick et al. 2017).

Disaster risk management entails comprehending the hazards and vulnerabilities of disasters, creating plans to lessen their effects, and effectively handling emergencies. Disaster risk is the interaction between natural hazards and the gradual deterioration of the exposed community’s vulnerability (Wisner et al. 2014). To grasp disaster risk correctly, it is vital to know not only the types of natural hazards but also the varying degrees of vulnerability of different groups of people, which are dictated by the community's socioeconomic system, power structure, and political practice (Wisner et al. 2014). By combining the potential roles of hazard, exposure, and vulnerability, the risk assessment demonstrates the likelihood of a system being affected by a disaster in the future. Risk assessment gives an awareness of the risk status of a community, allowing individuals to take action or implement mitigating measures to lower the predicted loss. Disaster risk combines hazard, exposure, and vulnerability, where a hazard is the kind, intensity, and frequency of a natural hazard process, and exposure is a spatial context that shows the likelihood of people and assets being affected by a particular hazard at a certain location. spatial context (Alam et al. 2020).

Applying the proper preventative steps might lessen the destruction caused by cyclones (Ahmed et al. 2016; Sattar and Cheung 2019). The results can inform effective cyclone mitigation methods of a thorough vulnerability assessment (Hoque et al. 2017, 2021). In the theoretical sense, vulnerability refers to the features and conditions of a community that make it susceptible to the adverse impacts of a hazard (UNDRR 2009). The term "vulnerability" refers to how susceptible a system is to the adverse effects of an environmental hazard in the context of climate change adaptation literature (Field 2014). Inequality, poverty, a dense population, a lack of resources, and a lack of education all contribute to an already vulnerable condition (Rana and Routray 2016). Thus, assessing a community's vulnerability to disasters is essential for reducing their catastrophic effects (Hoque et al. 2021; Ullah et al. 2021). One of the primary focuses of the 2015 Sendai Framework for Disaster Risk Reduction (SFDRR) and Sustainable Development Goals (SDGs) was on identifying, assessing, and reducing vulnerability in order to lessen disaster risk (SDGs 2015; SFDRR 2015).

There have been several attempts to examine vulnerability from diverse viewpoints and points of interest, including social vulnerability (Yoon 2012; Wisner et al. 2014), physical vulnerability (Thouret et al. 2014; Papathoma-Köhle et al. 2017), economic vulnerability (Briguglio 1995; Willroth et al. 2011), livelihood vulnerability (Hahn et al. 2009), infrastructural vulnerability (López-Martínez et al. 2017), institutional vulnerability (Rana and Routray 2018), attitudinal vulnerability (Birkmann et al. 2013), and environmental vulnerability (Marín-Monroy et al. 2020). Other researchers have made similar efforts to assess vulnerability in terms of exposure, sensitivity, and capability (Hahn et al. 2009; Birkmann et al. 2013; Zhou et al. 2015; Rana and Routray 2018). Recent changes in temperature and weather affect vulnerability because they present novel and diverse threats to our socioeconomic systems. As a result, it is crucial to reduce the vulnerability of individuals and communities to the impacts of extreme weather by increasing the prevalence of adaptation methods (Boero et al. 2015). The vulnerability has been calculated using several different models. Different stresses and disturbances are considered in the pressure and release model (Wisner et al. 2014). Vulnerability framework models consider how exposed, sensitive, and resilient a system is (Turner et al. 2003). The human vulnerability was defined under one paradigm as exposure, resistance, and resilience (Pelling 2003). The onion framework describes a vulnerability regarding how different hazards affect different parts of society and the economy (Birkmann 2006). An enhanced vulnerability assessment in Europe has been created using the MOVE framework (Birkmann et al. 2013). Quantitative vulnerability assessment based on indexes has gained traction recently (Tate 2012). A composite index is the most effective method for measuring the dynamic nature of vulnerability because it simplifies technical data for non-experts (Birkmann 2006). Key indicators must be identified, data must be standardized for comparison analysis, indicators must be weighted and aggregated, and uncertainty measures must be taken to assess and analyze the robustness of indicators; all of this can only be done within a conceptual framework (Adger et al. 2004).

Although Bangladesh is a cyclone-prone country, there are few vulnerability assessment studies (Hoque et al. 2021). Different studies looked into how adaptation and vulnerability to climate change affected Bangladesh (Huq et al. 1999; Yamin et al. 2005; Ahmed et al. 2019). Climate change has decreased agricultural output in Bangladesh's coastal area due to increased cyclones (Habiba et al. 2015; Ahmed et al. 2019). When people are unable to find legal ways to support themselves in the face of environmental pressures like cyclones, they may become even more vulnerable to these events (Ahmed et al. 2019). Some inhabitants of southwest coastal Bangladesh, who are at risk from climate-related disasters, refuse to leave their homes (Mallick et al. 2017). To put it simply, the lives and livelihoods of people living in coastal areas are in great danger due to poverty, unsustainable exploitation of natural resources, and frequent cyclones (Ashraful Islam et al. 2016). The study also found that small and marginal farmers in coastal areas face a decline in income (Jalal et al. 2021).

There are numerous strategies and protocols available for mitigating and preventing cyclone-related risks. These steps range from individual preparedness to large-scale infrastructure projects and require government, organization, and individual collaboration. The insurance sector and government agencies can use models to establish risk-consistent rates and conduct a cost–benefit analysis of mitigating measures (McAneney et al. 2016). Simple forethought and preparation can significantly lessen the risk of life and property loss. It includes safeguarding vital documents, preparing emergency packs, and, if necessary, evacuating (Usher et al. 2013). Activities that minimize greenhouse gas emissions can help lower the frequency and severity of cyclones and other extreme weather events (Shultz et al. 2018). Governments can establish Disaster Risk Reduction Management offices in each province, city, and municipality, as well as neighborhood and village committees (Alam and Ray-Bennett 2021). In addition, hazard mapping can assist in identifying locations vulnerable to cyclones, storm surges, and flooding (Akter and Dayem 2021). Providing cyclone shelters is one of the most effective ways to reduce the number of lives lost during cyclones. Since Bangladesh's independence in 1971, the government and international cooperation organizations have attempted to mitigate cyclone disasters by building early warning systems and constructing cyclone shelters (Miyaji et al. 2020). Creating coastal belts can help lessen the effects of cyclones (Nasreen et al. 2023).

This study intends to use the opportunity to look at vulnerability in two unions of Pathaghata Upazilla in the cyclone-prone Barguna district of Bangladesh. Another study was conducted among the Rakhain people, an ethnic minority, in the Barguna district (Rahman et al. 2022a) to evaluate vulnerability based on social, economic, physical, and institutional variables. The current study employed social, economic, physical, institutional, attitudinal, and environmental factors to evaluate the vulnerability of cyclones. We employed an index-based strategy to assess vulnerability. In order to collect data, a questionnaire survey was conducted. After that, statistical tests utilizing a standard scoring system were conducted to evaluate the area's vulnerability. The results of this research will be useful to policymakers at all levels of government in their efforts to create a generally applicable plan for reducing the threat of natural hazards in which all people may participate, regardless of their location, race, or other identifying characteristics.

## Methods

### Study area

The study was conducted in Patharghata Upazila of the Barguna district. With a population of around 893,000 and a density of 488 people per square kilometer, Barguna is one of Bangladesh's most rural districts (BBS 2013). It is approximately 247 km from the country's capital, Dhaka (RHD 2007; Rezwana and Pain 2021). Farmers make up 71.93% of Barguna's employment, and agricultural production is the primary economic activity (BBS 2013). Regarding socioeconomic status, the vast majority of people living there are impoverished (Health Bulletin 2013). This area has a high risk of damage from natural catastrophes, especially cyclones and coastal floods (Miah et al. 2004; Rahman et al. 2022a).

The region has been hit by several devastating cyclones, most recently in 2013 with Cyclone Mahasen and in 2016 with Cyclone Roanu (Rezwana and Pain 2021). Although Bangladesh: cyclone Yaas (2021) made landfall in India, it caused damage to the coast of Bangladesh. As a result, 1,300,000 people were affected, and 3 individuals died in Bangladesh. In addition, around 26,000 households and 39% of agricultural land were also destroyed in nine coastal areas (*Bangladesh: Cyclone YAAS* 2021). Barguna was one of the most impacted among them. Moreover, Cyclone Amphan (2020) partially damaged 263 hectares of land in this district, while 107 hectares were destroyed entirely (TBS Report 2020). Additionally, 4960 households were affected by Cyclone Roanu (2016). In addition, nearly 59% of the overall population of Barguna was affected by the 2013 cyclone Mahasen. As a result, 7 fatalities and 57% of agricultural land have been negatively harmed. In addition, around 7000 houses were severely damaged and a further 60,000 were slightly damaged (*Tropical Storm Mahasen: HCTT Phase 1 Joint Needs Assessment in Bhola, Barguna and Patuakhali Districts* 2013). However, cyclone Sidr destroyed most of the region's flood ridges in 2007, leaving people with the constant worry of having their homes swept away by the tides (Davidson 2008). For example, about 1335 people died, a total of 95,412 households were damaged, and 60–70% of agricultural land was destroyed (Tamima 2009; Dastagir 2015). Apart from this, cyclone Bulbul (2019), cyclone Feni (2019), cyclone Aila (2009) affected coastal regions of Bangladesh (Tribune Desk 2021). Table 1 summarizes the cyclone's impact in the Barguna district of Bangladesh.

**Table 1:** Impact of cyclone in Barguna, Bangladesh

Name of cyclones	Impact	Source
Cyclone Yaas (2021)	Severely impacted	(*Bangladesh: Cyclone YAAS* 2021)
Cyclone Amphan (2020)	-Partially damaged 263 hectares of land -Heavily damaged 107 hectares of land	(TBS Report 2020)
Cyclone Roanu (2016)	-Affected 4960 families	(Ahmed et al. 2016)
Cyclone Mahasen (2013)	-7 died -57% of agriculture land impacted -7000 houses heavily damaged -60,000 houses partially damaged	(*Tropical Storm Mahasen: HCTT Phase 1 Joint Needs Assessment in Bhola, Barguna and Patuakhali Districts* 2013)
Sidr (2007)	-Died 1335 people -Destroy 1,119.89 square kilometers - Ruined crop 60–70% -Houses damaged- 95,412 (Fully + Partially)	(Tamima 2009)

**Fig. 1:**
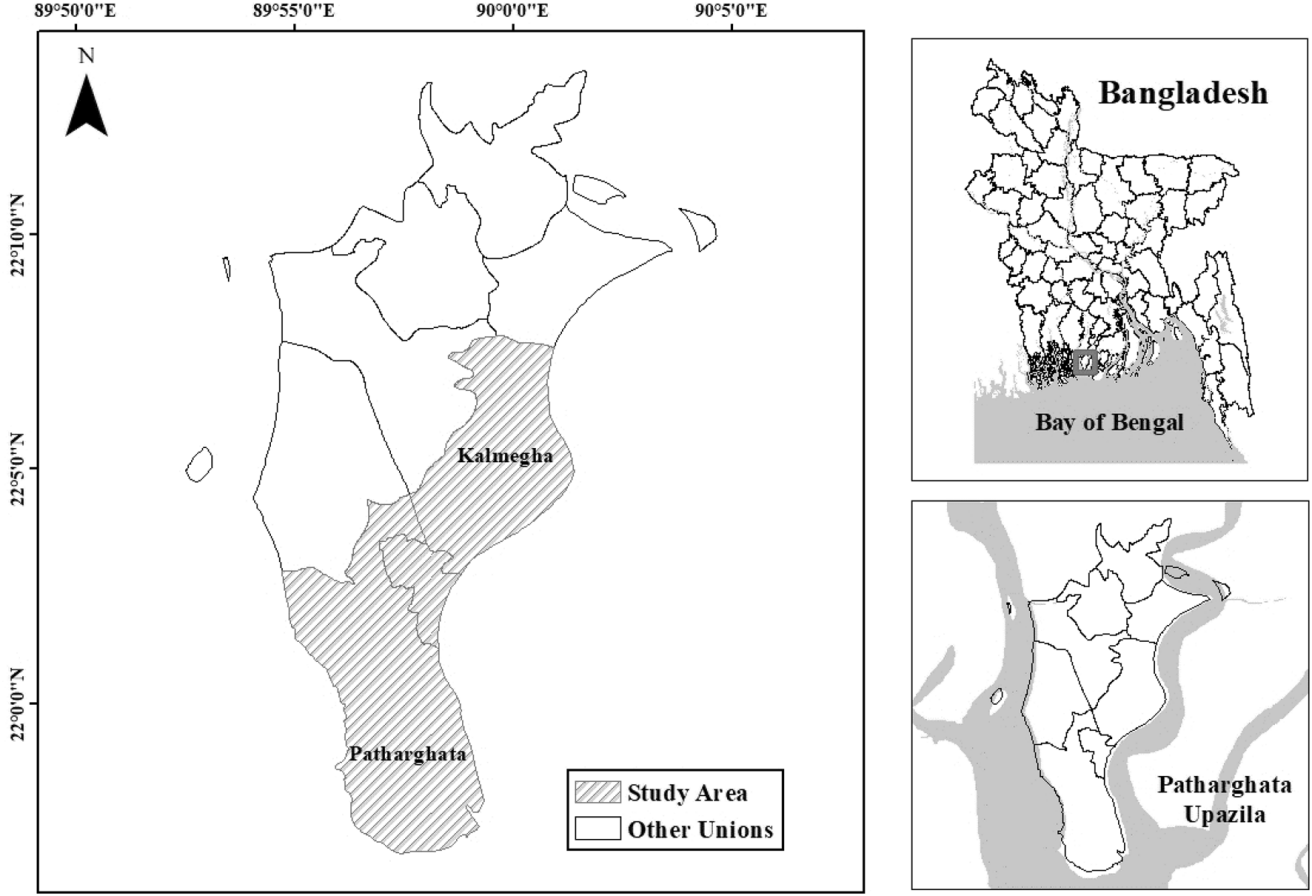
Study area (Source: Authors, 2022). Pathaghata and Kalmegha Unions are located in Patharghata Upazila, depicted by the right-side figures

Patharghata Upazila is a low-lying coastal area. It has a total land area of 387.36 square kilometers and a population of 1,63,927 (BBS 2013). The literacy rate in the region is 60.5%, and 423 people live there per square kilometer (BBS 2013). The Upazila is bounded by two rivers: the Baleshwari River on its west and the Bishkhali River on its east. The Bay of Bengal is situated in the south (Fig. 1). Patharghata Upazila comprises 7 unions: Raihanpur, Nachna Para, Patharghata, Kanthaltali, Kalmegha, Kakchira, and Char Duanti. Patharghata and Kalmegha are the largest, covering almost one-third of the total area (BBS 2013). Some of the unions in the Patharghata Upazila are located in a region with significant geophysical risk. The unions of Patharghata, Char Duanti, and Kalmegha are in an area of acute socioeconomic vulnerability, as shown by the Population and Structural Index (PSI), the Direct Access to Resources Index (DARI), and the Population Evacuation Need Index (PENI) (Tamima 2009). In terms of frequency and impact, the cyclone is the deadliest natural catastrophe in Patharghata (Islam et al. 2015). Thus, these regions are among the worst cyclone hits (Bangladesh Bureau of Statistics 2018).

### Data collection and data analysis

We conducted the households survey in April 2022 and asked each resident a series of predetermined questions. Six categories comprised the questionnaire: social vulnerability (9 indicators), economic vulnerability (8 indicators), physical vulnerability (10 indicators), institutional vulnerability (8 indicators), attitudinal vulnerability (5 indicators), and environmental vulnerability (3 indicators). First, we contacted neighborhood residents to see if they could help us gather information. Then, we selected some households where we could reach for data collection (based on convenience). We had a face-to-face survey with households. Therefore, we used a non-probability convenience sampling technique. This sampling technique was selected because it can be effective in some research scenarios, notably when time and resources are limited or when the target population is difficult to reach (Stratton 2021). Our study area was a remote location where it was challenging to reach the target population. 14,181 households live in Patharghata and Kalmegha Union (BBS 2013). Thus, 384 households were required following Morgan's table (95% Confidence Intervals (CI)) (Krejcie and Morgan 1970) for this perception-based study. The whole sample was divided evenly between the two unions using the proportional allocation method. As for the Kalmegha and Patharghata Union, we were able to contact 190 and 198 households, respectively.

We used the 'R' program, version 3.6.3 (R Development Core Team 2019), for statistical analysis. Where appropriate, descriptive statistics have been calculated. Following our previous study conducted among the Rakhian group (Rahman et al. 2022a), we also applied the chi-square test when comparing Kalmegha and Patharghata Union regarding vulnerability indicators. The data were not normally distributed, as the Shapiro–Wilk and Kolmogorov–Smirnov normality tests showed. When appropriate, the non-parametric Mann–Whitney U test was employed to evaluate the relationship between the Vulnerability Index Score (VIS) and the union. Similarly, we employed it in our earlier study examining COVID-19 responses in Bangladesh (Rahman et al. 2021) and fire preparedness in Dhaka City (Rahman et al. 2022b). When populations are not normally distributed, it is commonly employed as an alternative to the independent t-test. The 95% confidence level interval was used for all statistical analyses.

### Developing index

Indicators were selected after thoroughly reviewing the available literature (Cutter et al. 2003; Faruk et al. 2018; Masud-All-Kamal and Monirul Hassan 2018; Rana and Routray 2018; Maghfiroh and Hanaoka 2020; Marín-Monroy et al. 2020; Hoque et al. 2021; Ullah et al. 2021; Das et al. 2021; Noerhidajati et al. 2021; Rahman et al. 2022a). All indicators and their descriptions and sources are summarized in Table 2. Quantitative data for each indicator was gathered through a survey of households. A total of 43 were selected to measure household vulnerability. To better understand the nature of each indication, we placed them into several categories. After giving each indicator's class a score, the vulnerability index was calculated. In this analysis, we use Eq. 1 to determine how many scores each class of phenomena should receive on each indicator, and then we construct indexes based on those numbers. This study used a scoring allocation approach from a prior study (Ullah et al. 2021). In addition, we estimated the score based on our earlier evaluation of the Rakhain community's vulnerability in the Barguna district (Rahman et al. 2022a). Similar to earlier research, we aimed to maintain a score between 0 and 1. To do this, we assigned each indicator a score between 0 and 1 and then calculated the average value. Thus, we can compare the values of variables (including the composite vulnerability index) with the same score range.

1 $$Vulnerability\, Index(VI) = \,\sum\nolimits_{i = 1}^{n} {\frac{{S_{1} + S_{2} + S_{3} + \cdots S_{n} }}{n}}$$

where *S* = indicator’s corresponding score; *n* = the number of indicators.

**Table 2:** Explanation of the vulnerability indicators

Social vulnerability				
Indicators	Feature	Score	Explanation	Sources
Family size	Less than 4 4 to 6 More than 6	0 0.50 1	Larger families are assumed to be at greater risk	(Cutter et al. 2003; Birkmann et al. 2013; Rana and Routray 2018; Ullah et al. 2021)
Family type	Single Nuclear Extended	1 0.50 0	Human and social capital will make the extended family less vulnerable	(Rana and Routray 2018; Ullah et al. 2021)
Households with children	Yes No	1 0	Children and older people are vulnerable as they have limitations to movement and are weaker than younger people	(Hoque et al. 2021)
Households with older people (> 60 years)	Yes No	1 0		
Household head's educational attainment	No Primary Secondary School Higher Secondary and above	1 0.67 0.33 0	Higher-educated households have a greater comprehension of disaster preparedness, mitigation, and capacity building	(Rana and Routray 2018; Ullah et al. 2021)
Family members with higher education level (Higher Secondary and above)	Yes No	0 1		
Households residing periods in the community (in years)	Less than 10 10–20 20–30 30–40 More than 40	1 0.75 0.50 0.25 0	Long-term households are more knowledgeable about evacuation routes and local emergency procedures	(Rana and Routray 2018; Ullah et al. 2021)
Households with disabled members	Yes No	1 0	Disabled people have limitations in their daily activities, which makes them more vulnerable than ordinary people	(Hoque et al. 2021)
Households with chronically ill members	Yes No	1 0	People with chronic illnesses have limitations in their movement	(Hahn et al. 2009; Rana and Routray 2018)

Economic Vulnerability				
Indicators	Feature	Score	Explanation	Sources
Occupation of household head	Government/Private Job Trade and ommerce Agriculture Daily agers Unemployed	0 0.25 0.50 0.75 1	Insecure sources of income limit households pre, during, and post activities towards cyclones	(Phung et al. 2016; Mazumdar and Paul 2016; Rana and Routray 2018; Ullah et al. 2021)
A secondary source of income	Yes No	0 1	A household head with a secondary income source is considered less vulnerable	(Ullah et al. 2021)
Earning members of the households other than the household head	Yes No	0 1	Households with multiple earning members are less vulnerable	(Hahn et al. 2009; Rana and Routray 2018)
Dependency ratio (dependents to total household size)	Less than 0.41 0.41 to 1.34 1.35 to 2.29 More than 2.29	0 0.33 0.67 1	Due to their limited mobility and dependence, infants, children, and the elderly will be more at risk than young persons	(Phung et al. 2016; Rana and Routray 2018; Ullah et al. 2021)
Average annual household's income	Less than 50,000 50,000–100,000 100,000–150,000 More than 150,000	1 0.67 0.33 0	Low-income households would be more vulnerable, as they will have less capacity to recover from cyclones	(Cutter et al. 2003; Phung et al. 2016; Rana and Routray 2018; Ullah et al. 2021)
Outside-the-community-working family members	Yes No	0 1	During the cyclone, family members who work outside the neighborhood could assist the family physically, psychologically, and financially	(Hahn et al. 2009; Rana and Routray 2018)
Easily convertible (to cash) assets	Yes No	0 1	Households with productive assets are more financially strong as productive assets can turn into cash in need	(Flanagan et al. 2011; Rana and Routray 2018)
If a cyclone occurred today, would you be able to cover the costs?	Yes No	0 1	Households that believe they can manage costs if a hurricane strikes today appear to be psychologically and financially sound	(Flanagan et al. 2011; Rana and Routray 2018)

Physical vulnerability				
Indicators	Feature	Score	Explanation	Sources
Age of house (in years)	Less than 5 5–10 10–15 More than 15	0 0.33 0.67 1	Old houses are more vulnerable as they are structurally weaker	(Birkmann et al. 2013; Ullah et al. 2021)
Construction materials of household	Katcha (Tin-shed, Mud) Semi Pacca (Mixed with tin and brick) Pacca (Brick, Cement)	1 0.50 0	Household materials have a relation with vulnerability. For example, the vulnerability will be less if materials are strong (brick, cement). And tin-shed and mud make the household structurally vulnerable	(Mazumdar and Paul 2016; Rana and Routray 2018; Marín-Monroy et al. 2020; Ullah et al. 2021)
House elevation from flat land	Yes No	0 1	Elevated houses from flat land are considered safe from storm surges after cyclones	(Birkmann et al. 2013; Thouret et al. 2014)
Distance between households and nearest cyclone shelter (in km)	Less than 1 1–5 5–10	0 0.50 1	The greater the distance between the nearest cyclone shelter and households, the greater the vulnerability	(Sattar et al. 2020)
Distance between nearest medical facility and households (in km)	Less than 1 1–5 5–10	0 0.50 1	Households far from health care institutions require more time to get assistance, making them vulnerable	(Panthi et al. 2016; Ullah et al. 2021)
Condition of the closest cyclone shelter's WASH	Satisfied Neutral Dissatisfied	0 0.50 1	Despite cyclone shelters, the community is considered vulnerable if they are not well WASH facilitated	(Faruk et al. 2018)
Conditions of the nearest medical facility	Satisfied Neutral Dissatisfied	0 0.50 1	The better the condition of the nearest medical facility, the better the treatment	(Kawyitri and Shekhar 2021)
Household's access to proper sanitation	Yes No	0 1	Households that have access to proper sanitation will be less vulnerable	(Phung et al. 2016; Mazumdar and Paul 2016)
Electricity to the household	Yes No	0 1	Households with no electricity will suffer more in pre, post, and during phases of the cyclone	(Islam et al. 2013; Ullah et al. 2021)
Source of communication (Radio, TV, Mobile)	Yes No	0 1	Households with no source of communication are more vulnerable as they do not get information or cannot communicate with others	(Rana and Routray 2018; Ullah et al. 2021)

Institutional vulnerability				
Indicators	Feature	Score	Explanation	Sources
Understand early warning	Yes No	0 1	Institutions should ensure that the community understands the early warning	(Ahsan and Warner 2014)
Knowledge about cyclone	Yes No	0 1	Households with improper knowledge are considered vulnerable	(Ho et al. 2008; Ullah et al. 2021)
Knowledge about evacuation routes	Yes No	0 1	Households unaware of evacuation routes are considered vulnerable	(Rana and Routray 2018)
Frequency of public-awareness campaigns, exercises, and training	Often Rarely Never	0 0.50 1	Arranging frequent public awareness programs, drills, and training regarding cyclones demonstrate strong institutional behavior	(Rana and Routray 2018)
Received government relief after cyclone	Yes No	0 1	Cyclone vulnerability can be reduced by providing government assistance and humanitarian help	(Hossain 2020; Muñoz et al. 2021)
Received humanitarian aid from NGO/INGO after cyclone	Yes No	0 1		
Unequal relief distribution	Yes No	1 0	Inequitable relief and humanitarian aid distribution may exacerbate the vulnerability of households	(Maghfiroh and Hanaoka 2020)
Unequal humanitarian aid distribution	Yes No	1 0		

Attitudinal vulnerability				
Indicators	Feature	Score	Explanation	Sources
Community cooperation during cyclones	Poor Moderate Good	1 0.50 0	Community cooperation decreases vulnerability as community members can help each other during a disaster	(Panthi et al. 2016; Ullah et al. 2021)
Communication with local government over the year	Yes No	0 1	Households that have no connection with local government are considered vulnerable	(Hahn et al. 2009; Rana and Routray 2018)
Household feeling afraid of cyclones	Not worried at all Worried Very much worried	1 0.50 0	Households that do not feel afraid of cyclones will not get prepared for the future and might get vulnerable	(Ho et al. 2008; Pagneux et al. 2011)
Trust in government	Low Moderate High	1 0.50 0	Distrust in government may lead the community not to follow government initiatives	(Soane et al. 2010; Ullah et al. 2021)
Evacuation behavior during cyclone	Positive Negative	0 1	Negative evacuation behavior indicates more vulnerable	(Ahsan et al. 2016)

Environmental vulnerability				
Indicators	Feature	Score	Explanation	Sources
Source of drinking water	Ground water Surface water	0 1	Households with surface water to drink will be considered more vulnerable	(Rana and Routray 2018)
The salinity of the drinking water	Yes No	1 0	Households with access to drinkable water will be less vulnerable	(Hahn et al. 2009; Mazumdar and Paul 2016)
Trees can act as a natural barrier	Yes No	0 1	Cyclone speeds can be reduced by forestation, which in turn protects low-lying coastal areas	(Ataur Rahman and Rahman 2015; Younus 2017; Alam and Mallick 2022)

The above procedure calculates several vulnerability indices for each household in the study area. These indices include social vulnerability (SVI), economic vulnerability (EVI), physical vulnerability (PVI), institutional vulnerability (IVI), attitudinal vulnerability (AVI), and environmental vulnerability (EnVI). Using Eq. 2, we calculated a household's Composite Vulnerability Index (CVI). We have calculated the average CVI value to yield a score range of 0 to 1, similar to other variables. We can then compare CVI to other VI (which have the same score range). A previous study utilized a similar methodology (Ullah et al. 2021). In addition, we have applied it in our prior study on the Rakhain community (Rahman et al. 2022a).

$${\text{SVI}} = \,\sum\nolimits_{1}^{9} {\frac{{s_{i} }}{n}}$$

$${\text{EVI}} = \,\sum\nolimits_{1}^{8} {\frac{{s_{i} }}{n}}$$

$${\text{PVI}} = \,\sum\nolimits_{1}^{10} {\frac{{s_{i} }}{n}}$$

$${\text{IVI}} = \,\sum\nolimits_{1}^{8} {\frac{{s_{i} }}{n}}$$

$${\text{AVI}} = \,\sum\nolimits_{1}^{5} {\frac{{s_{i} }}{n}}$$

$${\text{EnVI}} = \,\sum\nolimits_{1}^{3} {\frac{{s_{i} }}{n}}$$

2 $${\text{CVI }} = \,\frac{{SVI{ + }EVI{ + }PVI{ + }IVI{ + }AVI{ + }EnVI}}{N}$$

where *i* represents the *i*th household and N the total number of vulnerability components.

In order to prevent over-valuation during the index calculation, we gave all indications the same score. For example, values between 0 and 1 were assigned to indicators with a dichotomous response, which only had two possible outcomes. Three responses were given scores of 0, 0.50, and 1. Similarly, each indicator for the five responses scored between 0.0 and 1.0. (Table 2). A value of 1.0 represents the most vulnerable category in the index, while a value of 0.0 represents the least vulnerable category.

### Ethical consideration

All the ethical guidelines outlined in the Declaration of Helsinki and its later revisions were strictly followed throughout this investigation involving human subjects (WMA 2018). Prior to each interview, informed consent was taken. In addition, this research has been approved by the Department of Disaster Management & Resilience, Bangladesh University of Professionals, Dhaka, Bangladesh, considering all the associated ethical issues.

## Results and discussion

### Overall vulnerability index

**Fig. 2:**
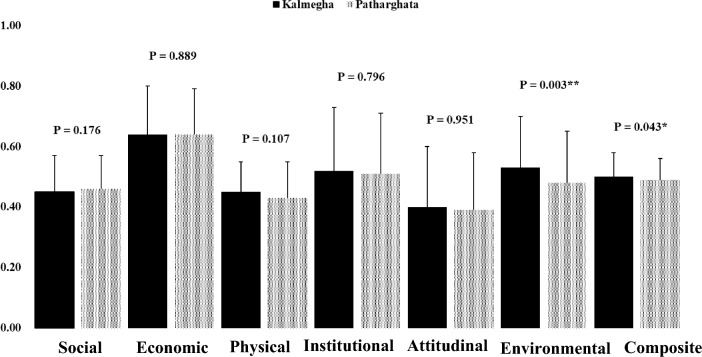
Mean and standard deviation of vulnerability index score based on areas

In the preceding paragraph, we went through each of the six vulnerability types in detail. Each area is more or less vulnerable to a certain range of components to its particular dynamics. When determining the Composite Vulnerability Index (CVI), we accounted for vulnerability across six categories: social, economic, physical, institutional, attitudinal, and environmental (Fig. 2 and Table 3). The CVI was determined using the methods discussed above. The average scores in the two categories of vulnerability—environmental and composite—are significantly different (Fig. 2). We have also estimated the standard deviation. Data with low standard deviation are grouped around the mean, whereas data with high standard deviation are more spread out. A standard deviation near zero means data points are close to the mean, while a high or low standard deviation means data points are above or below the mean. Kalmegha households are more environmentally vulnerable (0.53 ± 0.17), and they also show more composite vulnerability (0.50 ± 0.08) than their Patharghata counterparts. Half of the research regions indicated high environmental vulnerability, with Kalmegha showing far higher environmental vulnerability than Patharghata. Overall, people in Kalmegha are just as vulnerable as those in Patharghata, if not more so, when it comes to physical (0.45 ± 0.10), institutional (0.52 ± 0.21), and attitudinal (0.40 ± 0.20) vulnerability (Fig. 2). In both areas, the CVI score varied from a low of 0.25 to a high of 0.68. Table 3 shows that 19% of households in our survey region were in a particularly precarious situation. 23% of households in Kalmegha were highly vulnerable, compared to 15% in Patharghata (Table 3).

**Table 3:** Vulnerability index level

Vulnerability index (VI)	Overall *n* (%)	Kalmegha *n* (%)	Patharghta *n* (%)	*p*-value	Cramer’s V^#^
*Social Vulnerability Index (SVI)* High (> 0.53) Medium (0.39–0.53) Low (< 0.39)	83 (21.40) 186 (47.90) 119 (30.70)	42 (22.10) 83 (43.70) 65 (34.20)	41 (20.70) 103 (52.00) 54 (27.30)	0.221	0.09
*Economic Vulnerability Index (EVI)* High (> 0.76) Medium (0.55–0.76) Low (< 0.55)	90 (23.20) 199 (51.30) 99 (25.50)	41 (21.60) 99 (52.10) 50 (26.30)	49 (24.70) 100 (50.50) 49 (24.70)	0.755	0.04
*Physical Vulnerability Index (PVI)* High (> 0.52) Medium (0.38–0.52) Low (< 0.38)	90 (23.20) 189 (48.70) 109 (28.10)	43 (22.60) 101 (53.20) 46 (24.20)	47 (23.70) 88 (44.40) 63 (31.80)	0.169	0.09
*Institutional Vulnerability Index (IVI)* High (> 0.63) Medium (0.39–0.63) Low (< 0.39)	77 (19.80) 180 (46.40) 131 (33.80)	36 (18.90) 87 (45.80) 67 (35.30)	41 (20.70) 93 (47.00) 64 (32.30)	0.807	0.03
*Attitudinal Vulnerability Index (AVI)* High (> 0.50) Medium (0.31–0.50) Low (< 0.31)	87 (22.40) 141 (36.30) 160 (41.20)	44 (23.20) 67 (35.30) 79 (41.60)	43 (21.70) 74 (37.40) 81 (40.90)	0.896	0.02
*Environmental Vulnerability Index (EnVI)* High (> 0.66) Medium (0.34–0.66) Low (< 0.34)	0 (0.00) 197 (50.80) 191 (49.20)	0 (0.00) 111 (58.40) 79 (41.60)	0 (0.00) 86 (43.40) 112 (56.60)	0.004**	0.15
*Composite Vulnerability Index (CVI)* High (> 0.55) Medium (0.45–0.55) Low (< 0.45)	73 (18.80) 204 (52.60) 111 (28.60)	44 (23.20) 92 (48.40) 54 (28.40)	29 (14.60) 112 (56.60) 57 (28.80)	0.0834	0.11

Field survey, 2022 **p < 0.001. According to the relevant quartile value, the vulnerability index level was classified as high, medium, or low. Cramer’s V^**#**^** = **Cramer’s V (tables bigger than 2 × 2 contingency table) measures the strength of an association between two categorical variables (Akoglu 2018). Cramer’s V < 0.10 interprets weak, > 0.10 < 0.15 interprets moderate, > 0.15 < 0.25 strong, and > 0.25 very strong association (Akoglu 2018)

Additionally, in Kalmegha, the social vulnerability index (SVI) was between 0.74 and 0.13, while in Patharghata, it was between 0.75 and 0.14. Overall, around 21% of households were classified as highly vulnerable (Table 3). We looked at long-term residents in rural areas to see if they exhibit any defining social traits. Kalmegha's EVI varied from 0.13 to 0.97, and Patharghata's was between 0.16 and 0.93. About 23% of households were highly vulnerable in total. A comparison of the economic vulnerability index reveals that Patharghata is more economically vulnerable than Kalmegha, even though Kalmegha has more daily wagers and the jobless. Kalmegha may have had greater indices than Patharghata, including yearly revenue, more trade, commerce, etc.

The physical vulnerability index (PVI) scores fluctuated between 0.12 and 0.67 in Kalmegha and between 0.05 and 0.67 in Patharghata. About 23% of the population in the study region was highly physically vulnerable. As seen in Table 3, a number of variables point to a physical vulnerability, including the state of housing, the proximity to and quality of cyclone shelter, and the availability of medical care. The government should prioritize aiding these regions in gaining access to basic necessities. The study area's current infrastructure and essential services must be modernized to reduce its high vulnerability. In Kalmegha, the IVI was between 0 and 1, whereas in Patharghata, it was between 0.06 and 0.88. In addition, 20% of all households were highly vulnerable institutionally. It was also revealed that Pathaghata had a greater institutional vulnerability proportion than Kalmegha (Table 3). In Kalmegha, the AVI was between 0 and 1, whereas in Patharghata, it was between 0 and 0.80. Kalmegha had higher rates of attitudinal vulnerability than Patharghata.

### Vulnerability assessment

Six different types of vulnerability were measured. Many researchers have found that all six of these factors—social, economic, physical, institutional, attitudinal, and environmental—are interrelated (Cutter et al. 2003; Birkmann 2006; Birkmann et al. 2013; Ahsan and Warner 2014; Jamshed et al. 2017; Rana and Routray 2018; Ullah et al. 2021; Dintwa et al. 2022). We used the method described above to calculate the overall composite's vulnerability. The next section will discuss each vulnerability and the combined vulnerability of both areas. Class intervals for the individual and composite vulnerability indices were calculated using standard statistical methods.

#### Social vulnerability

The social vulnerability was measured using nine indicators derived from existing literature (Table 2). Comparing family sizes and residing periods in the community, a statistically significant *(p* < 0.05) difference was found between the two areas. One-third of all households had no more than four people living there, while another 57% had between four and six people living there, and only 8.50% had seven or more people living there (Table 4). There is a significant relationship between family size and vulnerability. Larger families are more at risk due to their larger size (Cutter et al. 2003; Rana and Routray 2018; Ullah et al. 2021). Having more family members may lead to an increased risk of the effects of cyclones (Rana and Routray 2018). Studies show that it becomes increasingly difficult after disasters to provide everyone's basic demands with limited financial resources (Cutter et al. 2003; Rana and Routray 2018; Ullah et al. 2021). It also depends on how old everyone is in the household. About 78% of families included children, whereas 33% included only those over 60 years. These older people and children around the house could need help during the cyclone. Fragile people, whether children or old, are especially vulnerable to the influence of a lack of physical and economic resources (Green et al. 1991; Morrow 1999; Hoque et al. 2021). Several studies have shown that the elderly suffer disproportionately from natural catastrophes (Lin et al. 2002; Jia et al. 2010; Alipour et al. 2015). Moreover, past research has shown that disasters have psychological consequences on children and the old (Kar 2009; Jia et al. 2010). Therefore, they require increased catastrophe relief (Green et al. 1991; Morrow 1999). It's worth noting that 92% of all households don't have any members who are disabled or chronically unwell (76%). This statistic suggests that most people living in a certain household were active and healthy. It is apparent in the routines of the rural population, where people are always doing something, be it farming, collecting a wage, or something else entirely. Households that include a person with a disability or a chronic illness will also be at a higher risk than those that do not. Therefore, they need outside help in times of catastrophe, such as cyclones.

**Table 4:** Vulnerability indicator results

Social vulnerability					
Indicators	Overall *n* (%)	Kalmegha *n* (%)	Patharghata *n* (%)	*p*-value	Cramer’s V or Phi (φ)^#^
Family size *Less than 4* *4 to 6* *More than 6*	131 (33.80) 224 (57.70) 33 (8.50)	64 (33.70) 100 (52.60) 26 (13.70)	67 (33.80) 124 (62.60) 7 (3.50)	0.001**	0.19
Family type *Single* *Nuclear* *Extended*	24 (6.20) 246 (63.40) 118 (30.40)	12 (6.30) 112 (58.90) 66 (34.70)	12 (6.10) 134 (67.70) 52 (26.30)	0.177	0.09
Households with children *Yes* *No*	304 (78.40) 84 (21.60)	152 (80.00) 38 (20.00)	152 (76.80) 46 (23.20)	0.443	0.04
Households with older adults (> 60 years) *Yes* *No*	128 (33.00) 260 (67.00)	68 (35.80) 122 (64.20)	60 (30.30) 138 (69.70)	0.298	0.06
Household head's educational attainment *No* *Primary* *Secondary School* *Higher Secondary and above*	181 (46.60) 164 (42.30) 27 (7.00) 16 (4.10)	87 (45.80) 82 (43.20) 10 (5.30) 11 (5.80)	94 (47.50) 82 (41.40) 17 (8.60) 5 (2.50)	0.243	0.10
Family members with higher education level (Higher Secondary and above) *Yes* *No*	66 (17.00) 322 (83.00)	32 (16.80) 158 (83.20)	34 (17.20) 164 (82.80)	1.00	0.00
Households residing periods in the community (in years) *Less than 10* *10 to 20* *20 to 30* *30 to 40* *More than 40*	28 (7.20) 39 (10.10) 63 (16.20) 110 (28.40) 148 (38.10)	8 (4.20) 11 (5.80) 28 (14.70) 47 (24.70) 96 (50.50)	20 (10.10) 28 (14.10) 35 (17.70) 63 (31.80) 52 (26.30)	0.000***	0.27
Households with disabled members *Yes* *No*	30 (7.70) 358 (92.30)	11 (5.80) 179 (94.20)	19 (9.60) 179 (90.40)	0.225	0.07
Households with chronically ill members *Yes* *No*	92 (23.70) 296 (76.30)	46 (24.20) 144 (75.80)	46 (23.20) 152 (76.80)	0.915	0.01
Economic Vulnerability					
Indicators	Overall *n* (%)	Kalmegha *n* (%)	Patharghata *n* (%)	*p*-value	Cramer’s V or Phi (φ)#
Occupation of household head *Government/Private Job* *Trade and commerce* *Agriculture* *Daily wagers* *Unemployed*	21 (5.40) 49 (12.60) 201 (51.80) 88 (22.70) 29 (7.50)	10 (5.30) 34 (17.90) 79 (41.60) 50 (26.30) 17 (8.90)	11 (5.60) 15 (7.60) 122 (61.60) 38 (19.20) 12 (6.10)	0.000***	0.22
A secondary source of income *Yes* *No*	38 (9.80) 350 (90.20)	17 (8.90) 173 (91.10)	21 (10.60) 177 (89.40)	0.705	0.03
Earning members of the households other than the household head *Yes* *No*	119 (30.70) 269 (69.30)	63 (33.20) 127 (66.80)	56 (28.30) 142 (71.70)	0.352	0.05
Dependency ratio (dependents to total household size) *Less than 0.41* *0.41 to 1.34* *1.35 to 2.29* *More than 2.29*	105 (27.10) 221 (57.00) 50 (12.90) 12 (3.10)	55 (28.90) 108 (56.80) 22 (11.60) 5 (2.60)	50 (25.30) 113 (57.10) 28 (14.10) 7 (3.50)	0.743	0.06
Average annual household's income *Less than 50,000* *50,000–100,000* *100,000–150,000* *More than 150,000*	48 (12.40) 109 (28.10) 136 (35.10) 95 (24.50)	21 (11.10) 51 (26.80) 70 (36.80) 48 (25.30)	27 (13.60) 58 (29.30) 66 (33.30) 47 (23.70)	0.762	0.05
Outside-the-community-working family members *Yes* *No*	35 (9.00) 353 (91.00)	18 (9.50) 172 (90.50)	17 (8.60) 181 (91.40)	0.898	0.01
Easily convertible (to cash) assets *Yes* *No*	214 (55.20) 174 (44.80)	102 (53.70) 88 (46.30)	112 (56.60) 86 (43.40)	0.639	0.03
If a cyclone occurred today, would you be able to cover the costs? *Yes* *No*	35 (9.00) 353 (91.00)	14 (7.40) 176 (92.60)	21 (10.60) 177 (89.40)	0.349	0.06
Physical Vulnerability					
Indicators	Overall *n* (%)	Kalmegha *n* (%)	Patharghata *n* (%)	*p*-value	Cramer’s V or Phi (φ)#
Age of house (in years) *Less than 5* *5 to 10* *10 to 15* *More than 15*	52 (13.40) 61 (15.70) 177 (45.60) 98 (25.30)	18 (9.50) 31 (16.30) 88 (46.30) 53 (27.90)	34 (17.20) 30 (15.20) 89 (44.90) 45 (22.70)	0.142	0.12
Construction materials of household *Katcha (Tin-shed, Mud)* *Semi Pacca (Mixed with tin and brick)* *Pacca (Brick, Cement)*	348 (89.70) 27 (7.00) 13 (3.40)	171 (90.00) 10 (5.30) 9 (4.70)	177 (89.40) 17 (8.60) 4 (2.00)	0.159	0.09
House elevation from flat land *Yes* *No*	382 (98.50) 6 (1.50)	188 (98.90) 2 (1.10)	194 (98.00) 4 (2.00)	0.685	0.04
Distance between households and nearest cyclone shelter (in km) *Less than 1* *1 to 5* *5 to 10*	148 (38.10) 228 (58.80) 12 (3.10)	54 (28.40) 124 (65.30) 12 (6.30)	94 (47.50) 104 (52.50) 0 (0.00)	0.000***	0.25
Distance between nearest medical facility and households (in km) *Less than 1* *1 to 5* *5 to 10*	63 (16.20) 286 (73.70) 39 (10.10)	14 (7.40) 165 (86.80) 11 (5.80)	49 (24.70) 121 (61.10) 28 (14.10)	0.000***	0.29
Condition of the closest cyclone shelter's WASH* Satisfied* *Neutral* *Dissatisfied*	153 (39.40) 91 (23.50) 144 (37.10)	73 (38.40) 57 (30.00) 60 (31.60)	80 (40.40) 34 (17.20) 84 (42.40)	0.007**	0.16
Conditions of the nearest medical facility *Satisfied* *Neutral* *Dissatisfied*	157 (40.50) 47 (12.10) 184 (47.40)	66 (34.70) 28 (14.70) 96 (50.50)	91 (46.00) 19 (9.60) 88 (44.40)	0.053	0.12
Household's access to proper sanitation *Yes* *No*	42 (10.80) 346 (89.20)	23 (12.10) 167 (87.90)	19 (9.60) 179 (90.40)	0.528	0.04
Electricity to the household *Yes* *No*	363 (93.60) 25 (6.40)	184 (96.80) 6 (3.20)	179 (90.40) 19 (9.60)	0.017*	0.13
Source of communication (Radio, TV, Mobile) *Yes* *No*	373 (96.10) 15 (3.90)	181 (95.30) 9 (4.70)	192 (97.00) 6 (3.00)	0.543	0.04
Institutional Vulnerability					
Indicators	Overall *n* (%)	Kalmegha *n* (%)	Patharghata *n* (%)	*p*-value	Cramer’s V or Phi (φ)#
Understand early warning *Yes* *No*	143 (36.90) 245 (63.10)	71 (37.40) 119 (62.60)	72 (36.40) 126 (63.60)	0.920	0.01
Knowledge about cyclone *Yes* *No*	58 (14.90) 330 (85.10)	22 (11.60) 168 (88.40)	36 (18.20) 162 (81.80)	0.093	0.09
Knowledge about evacuation routes* Yes* *No*	358 (92.30) 30 (7.70)	177 (93.20) 13 (6.80)	181 (91.40) 17 (8.60)	0.651	0.03
Frequency of public-awareness campaigns, exercises, and training *Often* *Rarely* *Never*	34 (8.80) 85 (21.90) 269 (69.30)	12 (6.30) 31 (16.30) 147 (77.40)	22 (11.10) 54 (27.30) 122 (61.60)	0.003**	0.17
Received government relief after cyclone *Yes* *No*	299 (77.10) 89 (22.90)	141 (74.20) 49 (25.80)	158 (79.80) 40 (20.20)	0.235	0.07
Received humanitarian aid from NGO/INGO after cyclone *Yes* *No*	243 (62.60) 145 (37.40)	120 (63.20) 70 (36.80)	123 (62.10) 75 (37.90)	0.915	0.01
Unequal relief distribution *Yes* *No*	276 (71.10) 112 (28.90)	132 (69.50) 58 (30.50)	144 (72.70) 54 (27.30)	0.552	0.04
Unequal humanitarian aid distribution *Yes* *No*	177 (45.60) 211 (54.40)	78 (41.10) 112 (58.90)	99 (50.00) 99 (50.00)	0.095	0.09
Attitudinal vulnerability					
Indicators	Overall *n* (%)	Kalmegha *n* (%)	Patharghata *n* (%)	*p*-value	Cramer’s V or Phi (φ)#
Community cooperation during cyclones *Poor* *Moderate* *Good*	90 (23.20) 56 (14.40) 242 (62.40)	39 (20.50) 35 (18.40) 116 (61.10)	51 (25.80) 21 (10.60) 126 (63.60)	0.069	0.12
Communication with local government over the year *Yes* *No*	187 (48.20) 201 (51.80)	102 (53.70) 88 (46.30)	85 (42.90) 113 (57.10)	0.044	0.11
Households feel afraid of cyclones *Not worried at all* *Worried* *Very much worried*	46 (11.90) 158 (40.70) 184 (47.40)	20 (10.50) 80 (42.10) 90 (47.40)	26 (13.10) 78 (39.40) 94 (47.50)	0.694	0.04
Trust in government *High* *Low* *Moderate*	83 (21.40) 220 (56.70) 85 (21.90)	30 (15.80) 115 (60.50) 45 (23.70)	53 (26.80) 105 (53.00) 40 (20.20)	0.031*	0.13
Evacuation behavior during cyclone *Positive* *Negative*	324 (83.50) 64 (16.50)	150 (78.90) 40 (21.10)	174 (87.90) 24 (12.10)	0.025*	0.12
Environmental Vulnerability					
Indicators	Overall *n* (%)	Kalmegha *n* (%)	Patharghata *n* (%)	*p*-value	Cramer’s V or Phi (φ)#
Source of drinking water *Ground water* *Surface water*	16 (4.10) 372 (95.90)	16 (8.40) 174 (91.60)	0 (0.00) 198 (100.00)	0.000***	0.21
The salinity of the drinking water *Yes* *No*	16 (4.10) 372 (95.90)	16 (8.40) 174 (91.60)	0 (0.00) 198 (100.00)	0.000***	0.21
Trees can act as a natural barrier *Yes* *No*	191 (49.20) 197 (50.80)	79 (41.60) 111 (58.40)	112 (56.60) 86 (43.40)	0.004**	0.15

Field survey, 2022 *p < 0.05, **p < 0.01, ***p < 0.001. Cramer’s V or Phi (φ)^#^ = Cramer’s V (tables larger than 2 × 2 contingency table) and Phi (2 × 2 contingency table) measure the strength of an association between two categorical variables (Akoglu^2018^). Cramer’s V and Phi < 0.10 interprets weak, > 0.10 < 0.15 interprets moderate, > 0.15 < 0.25 strong, and > 0.25 very strong association (Akoglu 2018)

We found that over half of the household, heads had no higher education credentials. Furthermore, 83% of households did not have a person with a higher secondary school degree or above. Due to the disparity in educational opportunities, both areas are more precarious than they could be. A lack of knowledge about early warning systems and emergency procedures can jeopardize households. Having more education makes one less vulnerable, whereas having less education makes one more so (Cutter et al. 2003; Rana and Routray 2018; Ullah et al. 2021). Those who have completed considerable education better understand the concepts of risk management, adaptability, and institutional strengthening. In addition, educational level is connected to socioeconomic status. It is often understood, for instance, that individuals with more education tend to make more money than those with less, which in turn influences various forms of vulnerability, such as physical and financial stability and more (Cutter et al. 2003; Rana and Routray 2018; Ullah et al. 2021). Despite this, studies show that traditional wisdom may also help lessen the disaster risk (Kelman et al. 2012).

Approximately 93% of the households had been there for more than 10 years, and 38% had been there for more than 40. The households' long histories in the area are evidence of the deep ties to the place felt by its current inhabitants. It demonstrates that most residents of the study region are aware of the threat. Residents learn more about local risks the longer they stay there. People who have lived in the area for a long time are likely to be deeply familiar with all it offers. When a cyclone or other disaster strikes, search and rescue efforts rely heavily on local expertise. Households' vulnerability decreases as their length of residence increases since they become familiar with the community's evacuation routes and safe zones (Ullah et al. 2021).

#### Economic vulnerability

Table 2 shows that eight indicators were used to evaluate economic vulnerability. The occupation of the household head was found to be significantly different between the two areas. Many households mainly relied on agriculture (52%) and daily wagering (23%). Agriculture was the main source of income for both Patharghata (62%) and Kalmegha Union (42%). People have lived in the study area for generations, and as we've already established, they're more likely to be landowners. Therefore, there will be less of a financial burden on those that own land in addition to their dwellings (land or a residence outside a cyclone-prone area) than there would be on those that did not. It shows that many Kalmegha households relied heavily on the availability of daily wage employment (26%) and the jobless (9%) to make ends meet, suggesting an unstable economic outlook. Barguna district's financial condition declined after Cyclone Sidr's 2007 destruction (Kabir et al. 2016).

About 90% of the households did not supplement their principal income with additional income sources. There was, however, no apparent distinction between the two areas in terms of alternative income sources (Table 4). The households in the area studied were especially economically vulnerable because they had limited access to alternative sources of income. Consequently, the introduction of alternative income-producing options would help reduce the vulnerability of the population in these two locations to a greater extent (Ullah et al. 2021). In addition, 31% of households included earners other than the head of the family. The dependence ratio, which measures how many people in a household rely on the household's income as a whole, was another key indication. It was determined by dividing the number of people below 18 years and above 60 years in the family by the number of people in the productive age range (18–60). In Comparison to Kalmegha (2.60%), Patharghata Sadar had a higher dependency ratio (3.50%).

Moreover, half of all households also have some sort of assets. For this reason, they can use these assets when they need to but don't have the money on hand. However, 91% of all households doubted their capacity to weather the cyclone's economic turmoil. The cyclone's impact compounded the economic precarity of already disadvantaged households. Similarly, the higher concentration of daily wagers and unemployment in Kalmegha demonstrates that its economy is more unstable than that of Patharghata. About 25% of Kalmegha's members claim to make more than 150,000 Bangladeshi Taka annually. Patharghata is the Sadar union (the main administrative union of Patharghata Upazilla). Comparing these two unions, Kalmegha may have better access to social and economic facilities owing to its proximity to the Paurasava area, where all the administrative institutions are located.

#### Physical vulnerability

There were ten measures used to evaluate physical vulnerability (Table 2). Physical vulnerability indicators in the research region varied significantly based on factors including the proximity to a cyclone shelter or medical facilities, the quality of the cyclone shelter's WASH facilities, and the prevalence of power in homes (Table 4). Many responded that the distance between their homes and the cyclone shelter or medical facilities was more than a kilometer. This finding suggests that these households were unable to get medical care promptly. That is indeed true; they will need to make the trip to see the doctor. Without access to healthcare, vulnerable populations, including children, the elderly, and expectant mothers, are at a greater risk of developing life-threatening conditions (Ullah et al. 2021). Adverse effects stemming from a lack of access to medical care were felt most keenly by those residing in rural regions. In addition, people's access to necessities is severely hampered since cyclones and storm surges damage roads and bridges. 37% of the respondents were dissatisfied with the WASH facilities of the cyclone shelter. The percentage of dissatisfied Patharghata people is far higher than that of dissatisfied Kalmegha locals. All people should be able to use cyclone shelters, regardless of socioeconomic status (Faruk et al. 2018). Additionally, about 89% of homes lacked access to adequate sanitary facilities. Sanitation services should be considered a basic human right alongside access to safe drinking water. Public health risks may arise if we are unable to control the situation. People may also be reluctant to move into the cyclone shelter because of a lack of appropriate sanitation. Contrarily, about 40% of the people in our sample were content with the local medical center. More importantly, it is a vital signal for determining whether or not to move cyclone shelters during times of emergency. Most residences have access to modern conveniences like electricity (93%). Compared to Patharghata Sadar (90%), Kalmegha (97%) has a higher electricity distribution. However, frequent maintenance is required to guarantee that this infrastructure is accessible to homes before, during, and after a cyclone or storm surge. The vast majority of homes (90%) in the region under study were Katcha (muddy houses). Only 3% of them were constructed using concrete. It suggests that the majority of the homes are potentially out of date due to their lack of contemporary brickwork and building designs. Households were more at risk from cyclone and storm surge damage because of a lack of government presence and the improper execution of construction rules. Constructing a home out of mud and wood leaves one extremely vulnerable to natural hazards like cyclones and storm surges. The 2007 cyclone was particularly destructive since nearly all Katcha shelters were entirely or partially destroyed, and all farmland was drowned (Younus 2017). Katcha houses had mud plinths (foundations). Therefore, two days of flooding ruined or degraded them (Younus 2017). Due to the high potential for cyclone and storm surge damage, authorities must closely monitor construction regulations. Over 95% of the population have access to at least one primary form of communication (TV, Radio, Mobile, etc.). These home-based means of communication are crucial in lowering vulnerability. For more effective early warning and information distribution, government and disaster management personnel should take advantage of accessible communication outlets in vulnerable communities.

#### Institutional vulnerability

The institutional vulnerability was assessed using eight indicators (Table 2). According to the findings, most households (92%) in the study area were aware of evacuation procedures in case of a cyclone. Nonetheless, over 80% and 60% lacked self-assurance in their understanding of cyclones and early warning systems. The key information source for individuals to use in developing a preparation strategy to lessen the impact of cyclones on their lives and livelihoods is cyclone early warning systems. Bangladesh has successfully implemented a cyclone preparedness program (CPP) (CPP, 2021). Over the past two decades, cyclone’s early warnings in Bangladesh have significantly reduced the number of cyclone-related fatalities (Ahsan et al. 2020). Existing early warning services nevertheless face several obstacles, requiring technological and non-technical enhancements (Ahsan et al. 2020). Thus, the appropriate authorities should set up an efficient cyclone early warning system in these cyclone regions. Campaigns to educate the public about the importance of an early warning system are also encouraged. In other words, the vulnerability may be considerably reduced with the help of a cyclone early warning system and public education campaigns. For the sake of people's safety and quick evacuations in the event of future cyclones, substantial efforts are required to expand their access to crucial information.

Approximately 77% and 62% of respondents got relief and aid, respectively, from the government and national and international organizations.; households in Patharghata received greater government relief (79%) than those in Kalmegha (74%). This result shows that aid workers might skip through Kalmegha in favor of Patharghata. It has been claimed that the Patharghata Union of the Patharghata Upazilla would offer better amenities. Authorities are tasked with strengthening public and private efforts to protect vulnerable members of all communities. As for the distribution of relief and humanitarian aid, 71% and 46% of households reported suffering unfairness. More than 65% of people in the study population had never participated in a cyclone-related public awareness program, such as a drill or training, despite living in one of the most cyclone-prone locations in the country. As a result, this finding may lend credence to the argument that a lack of knowledge about early warning systems necessitates more thorough cyclone drills. This indicator showed a significant difference between the two areas (Table 4). More than 75% of Kalmegha residents reported no community public awareness initiative in their area.

#### Attitudinal vulnerability

Five indicators were used to assess the attitudinal vulnerability (Table 2). Trust in government and evacuation behavior during cyclones significantly differed between the two areas (Table 4). Over half of them or so did not have faith in the government. As trust in government activities is substantially connected with successful response and recovery after cyclones, the relevant authorities should engage closely with the people and eliminate the trust gap. Kalmegha residents have far less faith in their government than in Patharghata. Nevertheless, 84% reported they acted positively during the cyclone evacuation, and residents in Patharghata displayed far more positive conduct than those in Kalmegha. More than 60% of the households surveyed believed they received strong community support throughout the cyclone. People in rural Bangladesh are known for their strong social bonding habits. Yet more than half of them need the local government to communicate with them since they are lacking this actively.

Around 47% of respondents are concerned about cyclones. Communities that are more likely to be severely impacted by future cyclones and more likely to be extremely worried about such events should be given priority when disaster risk measures are being planned. These findings suggest that the research area, particularly Kalmegha, is highly vulnerable to cyclones. For efficient cyclone risk management and communication, the government must forge close connections with the local populations.

#### Environmental vulnerability

Three indicators were used to assess the environmental vulnerability (Table 2). All three indicators show a statistically significant difference between the two areas (Tables 2, 3). The people in our sample group were found to be experiencing a drinking water shortage. 96% of the people surveyed in this research rely only on surface water. Even though few individuals noticed salinity in the drinking water, salinity has become a major issue in this area (Rahim et al. 2018; Islam et al. 2022). About half of those surveyed did not feel trees might serve as a natural barrier against cyclones and storm surges. After further discussion on this topic, we found that some people believed the trees could trigger more damage during cyclones. It should be noted that some naturally adapted plants and landscapes typically slow the velocity of cyclones and storm surges, so protecting the coastal zones and houses (Ataur Rahman and Rahman 2015; Younus 2017; Alam and Mallick 2022). Nonetheless, human activities have damaged plenty of forests and landscapes. Combining traditional and scientific management of coastal ecosystems with mangroves and other plants using an efficient method and habitat may mitigate the consequences of natural and climate-change-induced disasters, according to a study (Ataur Rahman and Rahman 2015).

## Conclusion

It is the first study to quantify cyclone vulnerability in rural Bangladesh. It's crucial to note that the study was done in a rural area; thus, the results may change in an urban situation. Thus, this study's findings should be interpreted cautiously. Maps that emphasize the data's decision-making utility are not evaluated here. Due to social indicators and psychological traits, vulnerability calculation might have limitations. In addition, the limitations might also be due to the time and effort necessary to construct the index and assign ratings. However, this study underscores the necessity to view vulnerability to cyclones and other natural hazards as a multidimensional process, including social, economic, physical, institutional, environmental, and attitudinal factors. It employs a tried-and-true approach for quantifying vulnerability by assigning each element a score. Using index-based indicators, we may determine the reasons for vulnerability and draw attention to them. This strategy can benefit from preparedness, risk reduction, recovery plans, and the oversight authority structure. This study indicated that communities in rural areas of Bangladesh are particularly at risk. It was found that the vulnerability patterns of the two regions are distinct. Both Kalmegha and Patharghata were revealed to be environmentally vulnerable. In terms of environmental and composite vulnerability, Kalmegha reveals a plethora of environmentally and socially fragile houses. The precarious conditions of respondents in the research area have been exacerbated by the region's unpredictable financial climate and deteriorating infrastructure. Before the cyclone hits, it is advised that disaster management officials in the affected district notify vulnerable populations. Similarly, while developing measures to mitigate the effects of natural catastrophes, governments should prioritize the most vulnerable populations. Local adaptation should also be assisted by government and aid organizations by providing alternatives and assistance. The study of vulnerability is a developing area that seeks to improve our comprehension of the underlying causes, resources, liabilities, and capacities involved with disasters. In the future, researchers, policymakers, and practitioners will be required to focus on a diverse array of issues pertaining to disaster risk. The establishment of a global vulnerability index to enable a better understanding of vulnerability in different regions of the world is one topic that must be addressed. This index could be a valuable tool for identifying vulnerable populations and designing interventions to reduce their vulnerability. Future research should also investigate the interactions and interrelationships between the key parts of a disaster system, such as environmental stability, hazard, and vulnerability. Such research can help us build more effective measures for disaster risk reduction by enhancing our understanding of the intricate relationships that influence the disaster system's function. In addition, future research should concentrate on mapping risks and vulnerabilities to improve planning for resilience. This may involve utilizing risk and capability evaluations to identify places and communities that are more prone to disasters, such as cyclones, flooding, fires, and earthquakes. Future research on vulnerability should also emphasize the creation of theoretical and modeling frameworks in order to better comprehend the interaction between disasters, vulnerability, and resilience. It may involve investigating how communities interact with risk and developing hypotheses to better comprehend the underlying reasons of vulnerability. This study expands the researcher's ability to implement CVI in other cyclone-prone regions. Depending on the targeted demographic, additional variables, such as cultural and political characteristics, can be included for a holistic vulnerability assessment.

## Data Availability

The data that support the findings of this study are available on request from the corresponding author.
